# Draft genome of *Lysinibacillus fusiformis* PwPw_T2 isolated from *Ananas comosus* revealing acetic acid producing and xenobiotic degrading enzymes

**DOI:** 10.1128/MRA.00753-23

**Published:** 2023-11-01

**Authors:** Oyetayo Olaoluwa Adefiranye, Adetomiwa Ayodele Adeniji, Olayide Folashade Obidi, Ganiyu Oladunjoye Oyetibo, Olubukola Oluranti Babalola

**Affiliations:** 1 Department of Microbiology, Faculty of Science, University of Lagos, Lagos, Nigeria; 2 Food Security and Safety Focus Area, Faculty of Natural and Agricultural Sciences, North-West University, Mmabatho, South Africa; Department of Biology, Queens College, Queens, New York, USA

**Keywords:** *Lysinibacillus*, xenobiotics, acetic acid, *Ananas comosus*, genome

## Abstract

*Lysinibacillus fusiformis* PwPw_T2 isolated from deteriorating *Ananas comosus* sample collected from Lagos State, Nigeria putatively possesses genomic features like potential enzymes catalyzing acetic acid production and xenobiotic compounds degradation via various pathways as indicated by its genome sequences. These could make the organism relevant in food waste valorization and micro-biotechnology.

## ANNOUNCEMENT

*Lysinibacillus fusiformis* PwPw_T2 was isolated from deteriorating *Ananas comosus* collected from Mushin Market, Lagos State, Nigeria (N 6^o^ 31’ 59.9988”, E 3^o^ 21’ 0”) as a potential vinegar producer while scavenging for acetic acid (vinegar) producers during food waste valorization. Before isolation, deteriorating *Ananas comosus* pulp was enriched on glucose, yeast extract, peptone, ethanol (GYPE) medium for 3 days followed by centrifugation at 250 rpm/2 min; pellets obtained were serially diluted. Dilutions 10^4^, 10^6^, and 10^8^ were inoculated on YCEA medium (yeast extract, calcium carbonate, and ethanol) for 3 days at 30°C. After observing visible zone of clearing, colonies were re-inoculated on YBE medium (yeast extract, bromocresol, and ethanol) to ascertain acetic acid production (1). Interest in organism’s genomic features has expanded (2–4). Hence, *L. fusiformis* PwPw_T2 genome was sequenced to further explore its genomic features and confirm presence of genes involved in acetic acid production.

Genomic DNA was extracted from fresh pure cultures on yeast extract peptone and glucose broth at 28°C–30°C for 48 hours with Quick-DNA fungal/bacterial miniprep kit (Zymo Research, Irvine, CA, USA) (5, 6). Library preparation and 2 × 150 bp paired-end sequencing were done with Nextera XT Index kit v2 (FC-131-2001 to -2004) and a NextSeq 500 system (Illumina, San Diego, CA, USA) at Quadram Institute Bioscience (Norwich, UK). Preassembly trimming was done by Trim Galore v0.6.5 (7). QUAST v5.0.2 was used to ascertain the quality of reads (8). The sequence reads were assembled into contigs with SPAdes v3.15.3 on KBase platform (9). Contigs from KBase platform were uploaded on online servers of NCBI prokaryotic Genome Automatic Annotation Pipeline (PGAAP v6.5), Bacterial and Viral Bioinformatics Resource Center (BV-BRC) (v3.30.19) (10), Rapid Annotations Subsystems Technology v2.0 (RAST), and SEED Viewer v2.0 (11, 12) for automated annotation and comparison. For all bioinformatics analyses, default settings were employed.

**Fig 1:**
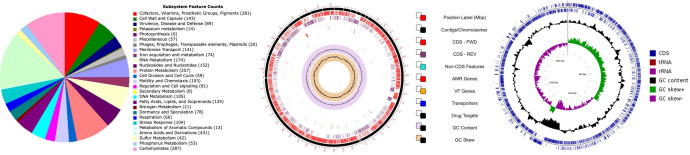
Overview of genome subsystems and circular views of *Lysinibacillus fusiformis* PwPw_T2 were created on RAST, Kbase, and BV-BRC online servers (9, 10, 12).

Details of *L. fusiformis* PwPw_T2 genome sequence are summarized in Table 1. The overview of subsystems unique to this bacteria’s genome is highlighted in Fig. 1. Based on putative genome exploration on BV-BRC/Pathosystems Resource Integration Center (PATRIC) online server, *L. fusiformis* PwPw_T2 genome possesses metabolic enzymes capable of degrading different types of xenobiotics via specific degradation pathway. Putatively, the isolate has potential for geraniol degradation by acetyl-CoA acetyltransferase [EC 2.3.1.9; pathway ID (PID) 00281] and fluorobenzoate degradation by N-acetylglucosamine deacetylase (EC 3.5.1; PID 00364). *L. fusiformis* PwPw_T2 also has the potential to biosynthesize alcohol dehydrogenase via glycolytic/glyconeogenetic pathway (EC 1.1.1.1; PID 00010) and aldehyde dehydrogenase via pyruvate metabolism (EC.1.2.1.3; ID 00620). Furthermore, in *L. fusiformis* PwPw_T2 genome, AntiSmash 7.0 run at default predicted biosynthetic clusters for the production of kijanimicin, beta-lactones, fengycin, petrobactin, terpenes, bacillibactin, and thiopeptides (13). The biosynthetic clusters of *L. fusiformis* PwPw_T2 make it a good candidate for further biotechnological manipulation, notably in the food manufacturing industry.

**TABLE 1:** Genome annotation features of *Lysinibacillus fusiformis* PwPw_T2^*a*^

Parameter	Value
Genome size	4,820,104 bp
Genes (total)	4,920
Number of contigs	21
Number of scaffolds	18
tRNA, rRNA	36, 5
GC percent	37
Hypothetical proteins	1,547
CDSs (total)^*b*^	4,874
CDSs (with protein)	4,824
Genes (RNA)	46
Contig N50	1.5 Mb
Contig L50	2

^*a*^ NCBI Prokaryotic Genome Automatic Annotation Pipeline (PGAAP v6.5) (https://www.ncbi.nlm.nih.gov/genome/annotation_prok/).

^*b*^ CDSs, coding sequences.

## Data Availability

The draft whole genome shotgun project has been deposited at DDBJ/ENA/GenBank under the accession number JAUIZN000000000.1. The version described in this paper is version JAUIZN010000000. The project data are available under BioProject accession number PRJNA991757 and BioSample accession number SAMN36317136 as well as Sequence Read Archive Accession number SRR25938945.
